# Effect of *Centella asiatica* on Oxidative Stress and Lipid Metabolism in Hyperlipidemic Animal Models

**DOI:** 10.1155/2014/154295

**Published:** 2014-04-16

**Authors:** Yun Zhao, Ping Shu, Youzhi Zhang, Limin Lin, Haihong Zhou, Zhentian Xu, Daqin Suo, Anzhi Xie, Xin Jin

**Affiliations:** ^1^Medical College, Xiamen University, Xiang'an District, Xiamen 361000, China; ^2^Xiamen Key Laboratory of Chiral Drugs, Xiamen 361000, China; ^3^College of Pharmacy, Hubei University of Science and Technology, Xianning 437000, China

## Abstract

Hyperlipidemia and many other metabolic diseases are related to oxidative stress. *Centella asiatica* is a traditional Chinese medicine whose antioxidant effect in vitro has been reported. We are interested in whether it possesses this effect in vivo and hence modulates lipid metabolism. Therefore, experiments were carried out on mice and golden hamsters regarding its antioxidant and hypolipidemic effect. We observed that a fraction (CAF3) of the ethanol extract (CAE) of *Centella asiatica* had a cholesterol decrease of 79% and a triglyceride decrease of 95% in acute mice model, so CAF3 was further investigated in high-fat-fed hamster model. It was shown that CAF3 increased SOD and GSH-Px activities and decreased MDA level, and it also improved TC, TG, LDL-C, HDL-C, AST, and ALT levels. L-CAT and SR-BI gene expression in hamsters were increased. Taken together, our data suggest that the CAF3 fraction of *Centella asiatica* has antioxidant and hypolipidemic properties.

## 1. Introduction

Oxidative stress has been shown to damage the structural and functional integrity of the cell either by directly modifying cellular DNA, proteins, and membrane lipids or by initiating chain reactions that cause extensive oxidative damage to DNA, proteins, and membrane lipids [1]. As for hepatocytes, once its functions are bad, the lipid metabolism is affected.

Abnormal lipid metabolism or hyperlipidemia, the high risk for atherosclerosis, is the condition that can result in many kinds of cardiovascular diseases [2]. Nowadays, medicinal plants play the key role in promoting human health and treating diseases. Traditional Chinese medicine has been reported to treat hyperlipidaemia and prevent atherosclerosis in many developing countries [3, 4]. As they are normally considered to be less toxic than synthetic agents, natural agents are increasingly purported to exert potent beneficial actions to regulate serum TC and TG levels and may thus play the role in reducing synthetic drug use for the treatment of hyperlipidemia [5]. Therefore, increasing interests have been drawn towards plant products [6].

*Centella asiatica* (L.) Urb. is a perennial herbaceous creeper of the Apiaceae family. It contains triterpenes, namely, asiatic acid (AA), madecassic acids (MA), asiaticoside (AD), madecassoside (MD) [7], essential oils, amino acids, and other compounds. In addition, it can cure some diseases by traditional treatment in China and other Asian countries, and its many pharmacological effects have been validated by modern technology, such as anti-inflammatory [8], memory improvement [9], anticancer [10], antihepatoma [11], antioxidation [12], and antigenotoxic [13]. The antioxidant capacity of* Centella asiatica* in vitro is also reported [14, 15]; however, there are few reports about antioxidant and hypolipidemic activities in vivo. Thus, the aim of the present study was to evaluate the effects of the* Centella asiatica* on oxidative stress and hyperlipidemia in hyperlipidemic mice and hamster models and to further explore the mechanism.

## 2. Materials and Methods

### 2.1. Chemicals and Reagents

Fenofibrate (Sigma-Aldrich, St. Louis, MO, USA) was dissolved in distilled water and administered by gavage to the mice at 100 mg/kg body wt (BW). Xuezhikang (Beijing Wbl Peking University Biotech Co., Ltd, China) was dissolved in distilled water and administered by gavage to the hamsters at 250 mg/kg body wt (BW). Triton WR-1339 (Tyloxapol, Sigma-Aldrich, USA) was dissolved in saline and administered by intraperitoneal injection to the mice at 400 mg/kg body wt (BW).

### 2.2. Plant Material and Extraction

*Centella asiatica* dry plant was obtained from LuYan Pharma Co., Ltd., Fujian Province of China and authenticated by Dr. Qiu, an expert in Medical College of Xiamen University, Xiamen, China. The concentrated solution (CAE) was prepared according to the following procedure: the powdered plant material (1 kg) was soaked in 8000 mL 95% ethanol and stand for 1 day, then extracted twice more in 95% ethanol at 80°C for 3 h. The filtered extract was concentrated by a rotary evaporator with a water bath at 60°C.

CAE was filtered again before being added to a HPD-450 macroporous resin (Cang Zhou Bon Adsorber Technology Co., Ltd., China) column, which was then washed sequentially with two column volumes of 50%, 70%, and 95% aqueous ethanol, designated CAF1 to CAF3, respectively. Each eluate was concentrated by vacuum rotary evaporation. The concentrated solution was dried in a lyophilizer and stored at 4°C until used. The yields of CAF1, CAF2, and CAF3 in terms of starting dried plant material were of 0.638, 0.676, and 0.824% (w/w).

### 2.3. Animals

Adult male KM mice (23–27 g) and male Golden Syrian hamsters (80–90 g) were used for the experiments. Mice and hamsters were obtained from SLAC Laboratory Animal Co., Ltd and Songlian farm of Songjiang District in Shanghai, respectively. The animals were housed under standard conditions of light and dark cycles with free access to food and water. All the animal studies were approved by the local ethics committee for animal experimentation (approval number XMULAC20120075) and were conducted under the National Institutes of Health Guidelines for the Care and Use of Laboratory Animals. All efforts were made to minimize the number of animals used and their suffering.

### 2.4. Experimental Design

#### 2.4.1. Triton WR-1339 Induced Hyperlipidaemia

Acute hyperlipidaemia, induced by intraperitoneal injection of Triton WR-1339, was used for the screening essay.

The mice were randomly divided into seven groups of 8 mice each. One group served as the normal control group (NCG) and another as the hyperlipidemia control groups (HCG) while the remaining 5 groups served as the medicated groups. NCG received intraperitoneal administration of normal saline and other groups of animals were treated with Triton WR-1339. NCG and HCG were treated with distilled water by intragastric administration. The medicated groups were treated with fenofibrate (HCG + Fen), CAE at 1500 mg/kg BW (HCG + CAE), CAF1 at 1000 mg/kg BW (HCG + CAF1), CAF2 at 1000 mg/kg BW (HCG + CAF2), and CAF3 at 1000 mg/kg BW (HCG + CAF3) by intragastric administration, respectively. After 12 hours, the animals were anaesthetized with 10% chloral hydrate (400 mg/kg). Blood samples were collected from their eyes and immediately centrifuged (3000 rpm/10 min). Serum was stored at −20°C for biochemical analysis.

#### 2.4.2. High-Fat-Fed Hyperlipidaemia

After 7-day accommodation, hamsters were randomly divided into 6 groups of eight animals each. The first group received distilled water and normal chow diet (NCD) and the second group was administered distilled water and high-fat diet (HFD) while animals in group III received standard drugs, Xuezhikang (250 mg/kg per day), and HFD (HFD + XZK). The group V-VII was treated with CAF3 (100, 250, and 500 mg/kg per day) and HFD (HFD + C100, HFD + C250, and HFD + C500, resp.). The high-fat diet consisted of 88% standard pellet diet, 10% lard, and 2% cholesterol. The blood samples were collected from the hearts of hamsters after 5 weeks of treatment. Livers were removed, cut into small portions, and stored at −20°C before use.

### 2.5. Biochemical Assays

Serum TC, TG, high density lipoprotein cholesterol (HDL-C), low density lipoprotein cholesterol (LDL-C), aspartate amino transferase (AST), and alanine amino transferase (ALT) levels were determined. The livers were analyzed regarding their superoxide dismutase (SOD), glutathione peroxidase (GSH-Px), and malondialdehyde (MDA). TC, TG, LDL-C, and HDL-C were estimated by using the enzymatic kits (Beijing BHKT Clinical Reagent Co., Ltd, China). AST, ALT, SOD, and MDA were assayed by commercially available kits (Nanjing jiancheng Bioengineering Institute, China). The liver index referred to the ratio of liver weight to animal weight and TC/HDL-C ratio calculated as the ratio of serum TC to HDL-C levels.

### 2.6. Histological Analysis

Liver tissues from hamsters experiment were fixed in 10% phosphate-buffered formalin for 1 day. Then liver slides (5 *μ*m thick) were cut with a cryostat microtome (Leica CM1950, Germany) and stained with hematoxylin and eosin (H&E) for microscopic examination.

### 2.7. Hepatic mRNA Expression of L-CAT and SR-BI

Total RNA was isolated from homogenized hamster livers by using TRIzol reagent (Invitrogen, Life Technologies, USA). Reverse transcriptions were performed in 20 *μ*L mixture with 1 *μ*g of total RNA according to the RT kit (MBI Fermentas, Canada). Primer sequences of hamster genes used in real-time PCR are listed as follows: L-CAT: 5′-TGGATGTGCTACCGTAAGACA-3′ (sense), 5′-TGTGGTTGTAGACAATCCTGGT-3′ (antisense) [16]; SR-BI: 5′-TTTGGAGTGGTAGTAAAAAGGGC-3′ (sense), 5′-TGACATCAGGGACTCAGAGTAG-3′ (antisense) [16], glyceraldehyde 3-phosphate dehydrogenase (GAPDH): 5′-GACCCCTTCATTGACCTCAAC-3′ (sen-se), 5′-GGAGATGATGACCCTTTTGGC-3′ (antisense) [17].

Real-time PCR was performed with the 7300 Real-Time PCR System (Applied Biosystems) and SYBR Green (Applied Biosystems) to measure gene expression. The cycling program was set as follows: thermal activation for 30 s at 95°C and 40 cycles of PCR (melting for 5 s at 95°C, followed by annealing/extension for 30 s at 60°C). LCAT and SR-BI gene expression data for individual samples were normalized to the corresponding GAPDH gene expression.

### 2.8. Analysis of AA, MA, AD, and MD in CAF3

AA, MA, AD, and MD in CAF3 were analyzed by high performance liquid chromatography (HPLC). The HPLC system was an Agilent, equipped with a UV detector at 205 nm. The standards, AA, MA, AD, and MD (Shanghai Yuanye Biotechnology Co., Ltd., China), and CAE, CAF1, CAF2, and CAF3 were dissolved in HPLC-grade methanol. The chromatographic analysis was performed at room temperature with a Cosmosil 5 C_18_-MS-II (250 mm × 4.6 mm) column using gradient elution of methanol (A) and water (B) according to the following profile for AA and MA: 60%–98% A of 0–25 min, 98% A of 25–35 min; the following profile for AD and MD: 20%–70% A of 0–30 min, 70%–98% A of 30–35 min. The flow rate was 1 mL/min.

### 2.9. Statistical Analysis

Results were presented as means ± SEM. Data were analyzed by one-way ANOVA test, followed by the Student-Newman-Keuls method. Differences were considered significant at *P* < 0.05. The Prism 5 software package (GraphPad Software Inc., USA) was employed for statistical tests and graphical presentation of the data.

## 3. Results

### 3.1. Effect of* Centella asiatica* Extracts on Triton WR-1339 Induced Hyperlipidemia

It was shown that Triton induced the elevation of TC and TG in serum compared to NCG. As shown in Table 1, it was demonstrated that the levels of TC and TG decreased significantly in the Fen, CAE, and CAF3 treatment groups, compared with the HCG. Among the three groups, the CAF3-treated group had the most significant effects on lowering TC and TG (*P* < 0.001).

### 3.2. Effect of CAF3 on High-Fat-Diet- (HFD-) Induced Hyperlipidemia

We next investigated the hypolipidemic effects of CAF3 in high-fat-diet- (HFD-) induced hyperlipidemic golden hamster model. After 35 days of high-fat intake, the hamsters gained body weight, liver weight, and liver index (Table 2), but treatment with XZK or CAF3 (100, 250, and 500 mg/kg) appreciably decreased the gain.

The serum lipid profiles of the hamsters in all groups are given in Table 3. In HFD group, serum TC and TG levels were increased by 307% and 181%, respectively. In comparison to the HFD, treatment with XZK and CAF3 (100, 250 and 500 mg/kg) significantly reduced serum TC by 42.5%, 51.8%, 61.3%, and 48.2% and TG by 31.6%, 46.7%, 57.9%, and 23.0%, while LDL-C by 45.0%, 38.4%, 62.0%, and 68.6%. There was no significant difference among HFD and treatment groups for serum HDL-C. For the ratio of TC to HDL-C, HFD hamsters produced a significant increase in this marker while the XZK and CAF3 (100, 250, and 500 mg/kg) reduced it in different degrees.

As shown in Table 4, serum ALT and AST levels were significantly lowered in hamsters fed different doses of CAF3 and XZK as compared with HFD (*P* < 0.001).

We also measured the antioxidant activity of CAF3. It was demonstrated in Table 4 that activities of SOD and GSH-Px, two key antioxidant enzymes, were increased, while those of MDA, an indicator of lipid peroxidation, were significantly decreased with treatment of XZK and CAF3 for 35 days (100, 250 and 500 mg/kg).

### 3.3. Pathologic Changes

**Figure 1:**
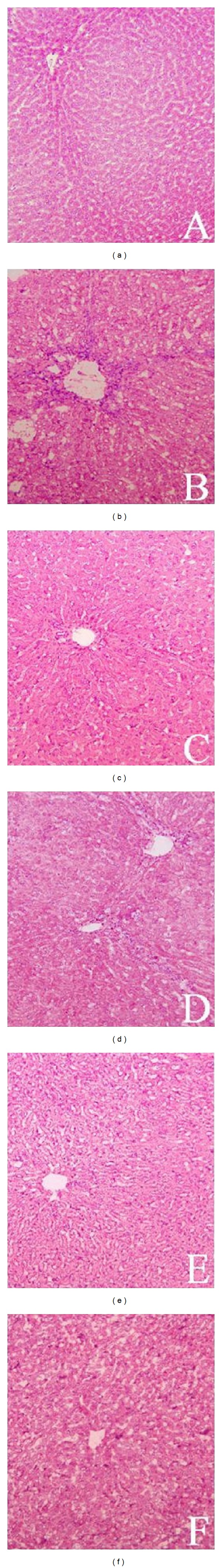
Histological examination detected by HE staining of golden hamsters liver frozen slices (magnification, ×100). (a) NCD; (b) HFD; (c) HFD + XZK (250 mg/kg) group; (d) HFD + C100 (100 mg/kg) group; (e) HFD + C250 (250 mg/kg) group; (f) HFD + C500 (500 mg/kg) group.

In order to examine the pathologic changes, we conducted the H&E staining of livers. It was revealed that the hepatocytes of animals in the NCD group had no hepatic steatosis, with normal hepatic sinusoids for mass transter (Figure 1(a)). But lipid deposits as macrovesicular and microvesicular steatosis were abundant in hamsters in HFD group, with damaged hepatic sinusoids (Figure 1(b)), compared to NCD group. It was also shown that treatment with XZK and different doses of CAF3 for 5 weeks substantially repressed these changes in HFD hamsters (Figures 1(c), 1(d), 1(e), and 1(f)). No necrotic cells or histologic evidence of hepatotoxicity was observed in standard or high-fat-diet-fed hamsters or any drug-treated hamsters.

### 3.4. Effect of CAF3 on LCAT and SR-BI mRNA Expression Levels

**Figure 2:**
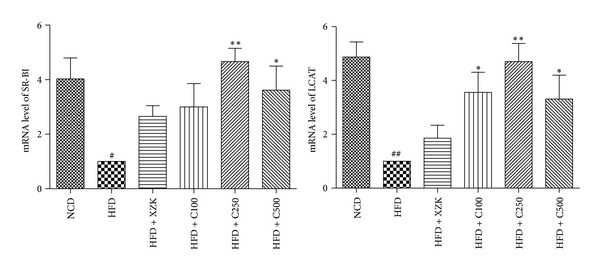
Effect of CAF3 on LCAT and SR-BI mRNA expression levels. A comparative threshold cycle (CT) method was used for relative quantification of gene expression using GAPDH for normalization. Measurements were carried out in triplicate for each sample. Data represents the mean of at least three independent experiments. ^#^ *P* < 0.05, ^##^ *P* < 0.01 compared to NCD. *P < 0.05, **P < 0.01 compared to HFD.

To better understand the mechanism of antihyperlipidemic activity of CAF3, we performed real-time PCR assay to investigate the mRNA expression of LCAT and SR-BI which play the key role in reversing cholesterol transport (RCT). It was revealed that the mRNA expression of LCAT and SR-BI was decreased in the hyperlipidemia model group, compared with NCD group (Figure 2). Both XZK and CAF3 at three different doses, with C250 having the best effect (*P* < 0.01), significantly elevated the LCAT and SR-BI mRNA levels compared with the HFD group, suggesting that LCAT and SR-BI were involved in the mechanism of lipid-lowering effect of CAF3.

### 3.5. Quantitative Determination of AA, MA, AD, and MD

Finally we conducted HPLC analysis to determine AA, MA, AD, and MD in CAF3, because they are the characteristic constituents in* Centella asiatica*. But the peaks of AD and MD were hardly separated from the peaks around them, so their data was not shown here. The result of AA and MA showed that the retention time of AA was 19.091 min and that of MA was 16.792 min. CAE, CAF1, CAF2, and CAF3, separately, showed a peak with the same wavelength and retention time of the AA and MA standard. The external standard method was used for quantification, and the result was shown in Table 5.

## 4. Discussion and Conclusions

Hyperlipidemia is characterized by excessive amounts of fatty substances such as cholesterol, triglycerides, and lipoproteins in the blood. The increase in the levels of the proteins and lipids can slow down metabolic processes because of blocked veins and arteries, which can result in cardiovascular disease [2]. The development of hyperlipidemia is related with high-fat diet and oxidative stress [18, 19]. There has been increasing interest in plants and natural products from them with respect to their antioxidant and lipid-lowering activity [6]. We use both acute and high-fat-fed hyperlipidemic models to illustrate the lipid-lowering effect of* Centella asiatica* and its antioxidant capacity in vivo.

The acute hyperlipidemic model was established by intraperitoneal injection of Triton WR-1339. Triton WR-1339 is a well-known nonionic detergent that has been used to induce acute hyperlipidemia in several animal models because of its ability to block TG-rich lipoprotein clearance and to increase hepatic cholesterol biosynthesis [20]. In our experiments, lipid concentrations (TC, TG, and LDL-C) in mice plasma were increased with Triton WR-1339 administration (Tables 2 and 3), similar to what was observed in other studies [21, 22]. Our result also demonstrated that CAF3 had the best effect on decreasing TG and TC level among all the* Centella asiatica *extracts and fractions. It suggests that the most effective compounds mainly were enriched in CAF3 by HPD-450 macroporous resin column chromatography.

The hypolipidemic effect of* Centella asiatica* was also estimated in high-fat-diet-induced hyperlipidemic hamster model. Hamsters have similar lipoprotein and bile acid metabolism patterns as humans [23]. We studied the effect of CAF3 on blood lipids of hamsters and it was shown that it lowered TC, TG, LDL-C, and TC/HDL-C (an indication of occurrence of coronary heart disease [24]) levels (Table 3). These results were consistent with those in the acute hyperlipidemia experiment in mice.

To explore the possible mechanism of hypolipidemic activity of* Centella asiatica*, we detected the gene expression of LCAT and SR-BI. LCAT is responsible for the synthesis of cholesteryl esters (CE) in human plasma [25]. It converts cholesterol into cholesteryl esters and may play the role in reversing cholesterol transport (RCT) [26]. LCAT activity was decreased in high-fat diet [27], and similar result has been reported in our study. SR-BI is a well-established HDL receptor and plays a key role in regulating plasma cholesterol levels [28]. Hepatic SR-BI expression is also an important positive regulator of RCT [29]. To conclude, LCAT and SR-BI jointly promote the process of RCT, which thus may play a role in reducing blood lipid and preventing hyperlipidemia. In the hamster study, we demonstrated that CAF3 enhanced the mRNA expression of LCAT and SR-BI in HFD-treated hamster livers. It indicates that the hypolipidaemic effect of CAF3 could be associated with upregulated RCT process.

Reactive oxygen species (ROS), commonly generated with a high-fat diet, have been observed in organs and tissues [30], as well as in the liver of the hyperlipidemic hamsters in our experiment. In our study, decreased SOD and GSH-Px and elevated MDA, which correlates with ROS, were detected when the hamsters were fed a high-fat diet (Table 4). As a result, hepatocytes of HFD hamsters were damaged, which was evident from elevated AST and ALT (Table 4) and H&E staining results of livers (Figure 1).

After treatment with CAF3 for 35 days, the hepatic levels of SOD and GSH-PX increased, and MDA decreased. This suggests that EPF3 has antioxidant capacity in vivo and hence protects the hepatocytes, which can tell from the reduced levels of AST and ALT (Table 4) and improved hepatic sinusoids (Figure 1). As the liver is in a better condition, it is able to regulate the metabolism of lipids more efficiently. This is confirmed by the reduction of the increased plasma lipid profile (Table 3).

It was reported that* Centella asiatica* is characteristic of some triterpenes, such as asiaticoside (AD), madecassoside (MD), asiatic acid (AA), and madecassic acid (MA) [31]. Therefore, we determined the contents of the four compounds in CAF3 to determine the active constituents against hyperlipaemia. The results showed that CAF3 contained the highest concentration of AA and MA, which happened to be reported to possess very good antioxidant capacity [14, 31]. AD and MD might also contribute to the hypolipidemic effect of* Centella asiatica*, but we cannot quantify them properly.

In conclusion,* Centella asiatica*, whose effective fraction is CAF3 and effective compounds are likely to be asiatic acid and madecassic acid, has antioxidant capacity both in vitro and in vivo, and it regulates lipid metabolism by antioxidant effect and LCAT and SR-BI enhancement. Taken together,* Centella asiatica* has the potential to be used for lipid regulation.

## Figures and Tables

**Table 1:** Effect of CA extracts on serum TC and TG levels in mice.

Groups	Dose (mg/kg d^−1^)	TC (mmol/L)	TG (mmol/L)
NCG	—	2.39 ± 0.09	0.26 ± 0.03
HCG	—	6.61 ± 0.62^##^	14.00 ± 1.67^###^
HCG + Fen	100	4.07 ± 0.70*	2.84 ± 0.68***
HCG + CAE	1500	2.55 ± 0.27**	4.69 ± 2.11***
HCG + CAF1	1000	4.95 ± 1.23	4.18 ± 1.56***
HCG + CAF2	1000	6.83 ± 1.13	6.99 ± 0.90***
HCG + CAF3	1000	1.39 ± 0.03***	0.65 ± 0.18***

Values are expressed as means ± SEM (standard error of mean) from eight animals in each group; NCG: normal control group; HCG: hyperlipidemia control groups; HCG + Fen: hyperlipidemic + fenofibrate group; HCG + CAE: crude extract group; HCG + CAF1–HCG + CAF3: hyperlipidemic + fractions group.

^##^ *P* < 0.01, ^###^ *P* < 0.001 compared to NCG.

*P < 0.05, **P < 0.01, ***P < 0.001 compared to HCG.

**Table 2:** Effect of CAF3 on body weight, liver weight, and liver index in hyperlipidemic golden hamsters.

Groups	Dose (mg/kg d^−1^)	Initial body weight (g)	Final body weight (g)	Liver weight (g)	Liver index (g/100 g)
NCD	—	87.00 ± 3.83	96.75 ± 8.97	3.27 ± 0.41	3.33 ± 0.13
HFD	—	87.14 ± 4.74	124.50 ± 4.635^#^	7.12 ± 0.32^###^	5.73 ± 0.22^###^
HFD + XZK	250	87.13 ± 2.70	101.50 ± 4.48*	5.76 ± 0.32*	5.38 ± 0.10
HFD + C100	100	86.25 ± 2.34	104.00 ± 4.04*	5.35 ± 0.27**	5.06 ± 0.21
HFD + C250	250	85.20 ± 2.19	85.00 ± 5.58***	4.24 ± 0.32***	4.47 ± 0.18***
HFD + C500	500	84.60 ± 2.90	93.75 ± 8.12*	5.25 ± 0.53**	5.59 ± 0.24

Values are expressed as means ± SEM from 8 animals each group; NCD: normal chow diet group; HFD: high-fat diet group; HFD + XZK: high-fat diet + Xuezhikang group; HFD + C100, HFD + C250, HFD + C500: high-fat diet + CAF3 (100, 250, 500 mg/kg) group, respectively.

^#^ *P* < 0.05, ^###^ *P* < 0.001 compared to NCD.

*P < 0.05, **P < 0.01, ***P < 0.001 compared to HFD.

**Table 3:** Effect of CAF3 on serum lipid profile in hyperlipidemic golden hamsters.

Groups	Dose (mg/kg d^−1^)	TC (mmol/L)	TG (mmol/L)	HDL-C (mmol/L)	LDL-C (mmol/L)	TC/HDL-C
NCD	—	1.75 ± 0.15	0.54 ± 0.10	1.16 ± 0.19	2.55 ± 0.25	1.71 ± 0.26
HFD	—	7.13 ± 1.15^###^	1.52 ± 0.16^###^	3.22 ± 0.28^###^	9.68 ± 1.33^###^	2.46 ± 0.22^#^
HFD + XZK	250	4.10 ± 0.32***	1.04 ± 0.07*	2.70 ± 0.17	5.32 ± 0.26***	1.66 ± 0.19*
HFD + C100	100	3.44 ± 0.16***	0.81 ± 0.08***	2.75 ± 0.28	5.96 ± 0.51***	1.40 ± 0.20**
HFD + C250	250	2.76 ± 0.18***	0.64 ± 0.05***	2.93 ± 0.15	3.68 ± 0.33***	0.91 ± 0.08***
HFD + C500	500	3.69 ± 0.61***	1.17 ± 0.21*	2.60 ± 0.13	3.04 ± 0.12***	1.35 ± 0.21**

Values are expressed as means ± SEM from 8 animals each group; NCD: normal chow diet group; HFD: high-fat diet group; HFD + XZK: high-Fat diet + Xuezhikang group; HFD + C100, HFD + C250, HFD + C500: high-fat diet + CAF3 (100, 250, 500 mg/kg) group, respectively.

^#^ *P* < 0.05, ^###^ *P* < 0.001 compared to NCD.

*P < 0.05, **P < 0.01, ***P < 0.001 compared to HFD.

**Table 4:** Effect of EPF3 on liver in hyperlipidemic golden hamsters.

Groups	Dose (mg/kg d^−1^)	ALT (U/L)	AST (U/L)	SOD (U/mgprot)	GSH-Px (U/mgprot)	MDA (nmol/mgprot)
NCD	—	24.61 ± 8.81	17.01 ± 2.87	38.45 ± 5.54	3819 ± 305	0.46 ± 0.01
HFD	—	117.8 ± 29.09^###^	71.36 ± 12.15^###^	20.42 ± 1.41^##^	2160 ± 113^###^	0.62 ± 0.04^###^
HFD + XZK	250	31.58 ± 5.87***	23.41 ± 1.78***	32.08 ± 1.35*	3044 ± 122*	0.35 ± 0.02***
HFD + C100	100	21.55 ± 5.50***	20.09 ± 2.56***	27.62 ± 1.78	3272 ± 125*	0.35 ± 0.01***
HFD + C250	250	20.20 ± 3.15***	16.29 ± 1.73***	31.57 ± 3.26*	3693 ± 103**	0.40 ± 0.01***
HFD + C500	500	39.64 ± 13.05***	27.44 ± 4.06***	38.15 ± 2.72**	2610 ± 100	0.40 ± 0.01***

Values are expressed as means ± SEM from 8 animals each group; NCD: normal chow diet group; HFD: high-fat diet group; HFD + XZK: high-fat diet + Xuezhikang group; HFD + C100, HFD + C250, HFD + C500: high-fat diet + CAF3 (100, 250, 500 mg/kg) group, respectively.

^##^ *P* < 0.01, ^###^ *P* < 0.001 compared to NCD.

*P < 0.05, **P < 0.01, ***P < 0.001 compared to HFD.

**Table 5:** Content of AA and MA in CAE and different fractions.

Content (w/w)	CAE	CAF1	CAF2	CAF3
AA	4.28%	1.35%	1.69%	7.64%
MA	3.36%	1.20%	1.35%	7.68%
